# Multiplexing PKA and ERK1&2 kinases FRET biosensors in living cells using single excitation wavelength dual colour FLIM

**DOI:** 10.1038/srep41026

**Published:** 2017-01-20

**Authors:** Claire Demeautis, François Sipieter, Julien Roul, Catherine Chapuis, Sergi Padilla-Parra, Franck B. Riquet, Marc Tramier

**Affiliations:** 1 CNRS, UMR 6290, Rennes, France; 2 Université de Rennes 1, Institut de Génétique et Développement de Rennes, France; 3 Molecular Signaling and Cell Death Unit, Department of Biomedical Molecular Biology, Ghent University, Ghent, Belgium; 4 Molecular Signaling and Cell Death Unit, Inflammation Research Center (IRC), VIB, Ghent, Belgium; 5 Equipe Biophotonique Cellulaire Fonctionnelle, Laboratoire de Physique des Lasers, Atomes et Molécules (PhLAM), CNRS-UMR 8523, Villeneuve d′Ascq, France; 6 Division of Structural Biology, University of Oxford, The Henry Wellcome Building for Genomic Medicine, Headington, Oxford OX3 7BN, UK; 7 Cellular Imaging Core, Wellcome Trust Centre for Human Genetics, University of Oxford, UK; 8 Unité de Glycobiologie Structurale et Fonctionnelle, CNRS-UMR 8576, Université Lille1, Villeneuve d′Ascq, France; 9 Microscopy Rennes Imaging Centre, Biosit, Université de Rennes 1, France

## Abstract

Monitoring of different signalling enzymes in a single assay using multiplex biosensing provides a multidimensional workspace to elucidate biological processes, signalling pathway crosstalk, and determine precise sequence of events at the single living cell level. In this study, we interrogate the complexity in cAMP/PKA-MAPK/ERK1&2 crosstalk by using multi-parameter biosensing experiments to correlate biochemical activities simultaneously in time and space. Using a single excitation wavelength dual colour FLIM method we are able to detect fluorescence lifetime images of two donors to simultaneously measure PKA and ERK1&2 kinase activities in the same cellular localization by using FRET biosensors. To this end, we excite two FRET donors mTFP1 and LSSmOrange with a 440 nm wavelength and we alleviate spectral bleed-through associated limitations with the very dim-fluorescent acceptor ShadowG for mTFP1 and the red-shifted mKate2 for LSSmOrange. The simultaneous recording of PKA and ERK1&2 kinase activities reveals concomitant EGF-mediated activations of both kinases in HeLa cells. Under these conditions the subsequent Forskolin-induced cAMP release reverses the transient increase of EGF-mediated ERK1&2 kinase activity while reinforcing PKA activation. Here we propose a validated methodology for multiparametric kinase biosensing in living cells using FRET-FLIM.

External signals are integrated at the cellular level through a cascade of events that amplifies and transmits the incoming signal towards a specific outcome^1^. To regulate intensity, duration and specificity of the response, a cell relies on several interconnected signal transduction pathways^2^. This phenomenon, referred to as crosstalk, is defined by the orchestrated action of effector molecules to elicit a specific cellular response^3^. A typical example of signalling pathways crosstalk is that of cyclic adenosine monophosphate/protein kinase-A (cAMP/PKA) with the mitogen-activated protein kinase/extracellular signal-regulated kinase 1&2 (MAPK/ERK1&2)^4^.

The serine/threonine kinase ERK, one member of the MAPK family, propagates a variety of cellular activities depending on its spatiotemporal activation state. The MAPK cascade is composed of three-layers of kinases acting as a signalling relay: a MAPK kinase kinase (Raf), a MAPK kinase (MEK) and a MAPK (ERK)^5^. Another layer of regulation of the pathway involves scaffold proteins as well as positive and negative feedbacks to control the duration, the magnitude and subcellular compartmentalization of ERK activity^6^,^7^,^8^. PKA is a serine/threonine kinase that forms a tetramer in its inactive state containing two regulatory (R) and two catalytic (C) units. Upon binding of cAMP to the two regulatory subunits, the two catalytic subunits dissociate from the holoenzyme and then become active^9^. Once activated, both PKA and ERK1&2 translocate into the nucleus to phosphorylate numerous downstream substrates. Although first described more than 20 years ago and well documented since, it is still intensively studied, as there are still some conflicting results and unresolved issues^10^,^11^. In fact, combinatorial possibilities, expression patterns, activity profiles and spatiotemporal regulation of cell- and situation- specific effector molecules are complicating the analyses of dynamic signalling networks.

The best-characterized connections are PKA-dependent ERK activity modulation mediated by different Raf isoforms in different cell types^11^. These differential effects are explained by the fact that Raf kinase family comprises different isoforms. These carry out non-redundant functions, are not ubiquitously found in all tissue, and can be expressed at different levels^12^,^13^. PKA scaffold proteins such as the A-kinase anchoring proteins (AKAP) together with that of ERK1&2, the kinase suppressor of Ras (KSR), can enhance cAMP control of the ERK1/2 cascade^14^,^15^ regulating PKA and ERK1&2 activation as well as phosphodiesterases (PDE) by controlling intracellular cAMP levels^14^,^16^. Another connection is found at the level of PDEs, which terminate cAMP signalling by hydrolysing cAMP. PDEs that are directly targeted by ERK1&2^17^ were recently found to bind to and to regulate Raf-1 kinase^18^.

Such a level of complexity in cAMP/PKA-MAPK/ERK1&2 crosstalk calls for novel approaches. Genetically encoded Förster Resonance Energy Transfer (FRET) biosensors are powerful tools for monitoring spatiotemporal biochemical activities in living samples^19^. The main advantage of these tools is to monitor the amplitude, the duration and the localization of a biochemical activity during time-lapse fluorescence microscopy acquisition in living samples. Multi-parameter biosensing experiments have become essential to correlate biochemical activities during a dedicated cellular process. A very exciting challenge has thus been to follow several FRET biosensors in the same sample at the same time and location^20^,^21^,^22^,^23^,^24^. Although simultaneous recording of multiple cellular events was managed, the multiplex FRET biosensors approaches suffer from two limitations: (i) a spectral bleed-through of the first acceptor in the second donor emission band dependent on biosensors concentration, and (ii) the multiple excitation wavelengths necessitate sequential acquisitions that are not optimal for simultaneous observation of several biosensors. Here, we report on a method dealing with these different limitations for multiplexing genetically encoded FRET biosensors. We reasoned that single excitation wavelength combined with FRET-FLIM measurements could overcome such limitations. Taking advantage of the long stoke shift of Large Stoke Shift mOrange (LSSmOrange)^25^, we used a 440 nm single excitation wavelength for both donors, monomeric teal fluorescent protein (mTFP1) and LSSmOrange, and a dual colour fluorescence lifetime imaging microscopy (FLIM) to simultaneously measure signals from two genetically encoded FRET biosensors. We took advantage of dim-fluorescent acceptors; a yellow fluorescent protein (YFP)-based resonance energy accepting chromoprotein (sREACh) and its blue shift spectrum mutant (ShadowG)^26^ for mTFP1, and the red-shifted mKate2^27^ for LSSmOrange. Because ShadowG is darker than sREACh^26^ (which is one hundred fold less fluorescent than YFP) the spectral bleed-through between the two biosensors was reduced. We validated our approach by applying this methodology to simultaneously monitor, for the first time, two kinase activities at the same cellular location. MAPK/ERK1&2 and cAMP/PKA interplay in living HeLa cells was examined using EKAR2G^28^ and AKAR4^29^ biosensors respectively modified with mTFP1/ShadowG and LSSmOrange/mKate2 fluorescent protein pairs. We could report on EGF-mediated ERK1&2 and PKA activation in HeLa cells. The relevance of our methodology was clearly illustrated upon Forskolin-mediated increase in cAMP levels following EGF stimulation. Using these conditions we could simultaneously record cAMP inhibitory effect on EGF-mediated ERK1&2 activity level together with its reinforcing of PKA activity.

## Results

### LSSmOrange is an appropriate FRET donor for mKate2 and can be simultaneously used with mTFP1 in single excitation wavelength dual colour FLIM

**Figure 1:**
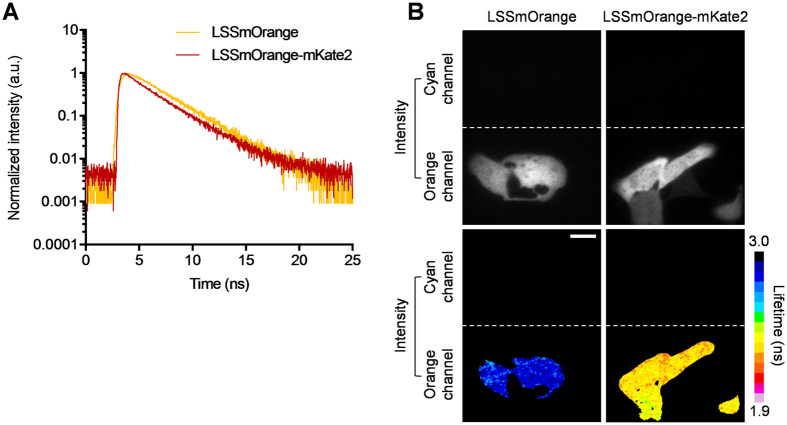
LSSmOrange has a mono-exponential decay and forms with mKate2 an effective FRET pair. (**A**) Representative LSSmOrange fluorescence lifetime decay profiles measured by TCSPC in U2OS cells expressing either LSSmOrange or LSSmOrange-mKate2 tandem constructs. The LSSmOrange-mKate2 decay has a shorter slope than that of LSSmOrange. LSSmOrange shows a mono-exponential decay while LSSmOrange-mKate2 tandem harbors a bi-exponential decay. LSSmOrange alone: τ = 2.75 ± 0.07 ns (n = 11) from mono-exponential fit; LSSmOrange-mKate2 tandem: τ_1_ = 2.80 ns (fixed), τ_2_ = 1.18 ± 0.12 ns and a_2_/a_1_ + a_2_ = 0.54 ± 0.02 (n = 12). (**B**) Representative intensity and fluorescence lifetime images of either LSSmOrange or LSSmOrange-mKate2 tandem, expressed in U2OS cells, acquired with the fastFLIM system. The orange channel provides either intensity or fluorescence lifetimes: for LSSmOrange: τ_LSSmOrange_ = 2.76 ± 0.03 ns (n = 34) and LSSmOrange-mKate2: τ_LSSmOrange-mKate2_ = 2.32 ± 0.08 ns (n = 56). Note that LSSmOrange fluorescence intensity was only detectable in the orange channel. Fluorescence lifetimes were calculated as a mean ± SD. (n) indicates number of cells from at least 3 independent experiments. Scale bar = 10 μm.

To complement a conventional cyan FRET donor, we first searched for a second FRET donor that could also be excited at 440 nm but with a red-shifted emission spectrum to accommodate simultaneous acquisition of the two donors. We used Large Stoke Shift mOrange (LSSmOrange) that is excited at 440 nm and emits in the orange at 572 nm^25^. To validate our choice, we needed to determine whether LSSmOrange was suitable for FRET-FLIM experiments. Fluorescence decay measurements in U2OS cells expressing LSSmOrange were performed by TCSPC. LSSmOrange fluorescence decay can be best fitted with a single exponential model, with a measured lifetime of 2.75 ± 0.07 ns (Fig. 1A).

While mKate2 was already successfully used as an acceptor for LSSmOrange for intensity-based FRET studies^25^, we verified that this is also the case for FRET-FLIM. LSSmOrange and LSSmOrange-mKate2 tandem plasmids were transfected in U2OS cells and cells were subjected to FRET-FLIM measurements using both (TCSPC) and time gated FLIM systems^30^. LSSmOrange-mKate2 tandem showed a faster decay as compared to LSSmOrange alone (Fig. 1A). This difference in fluorescence decay indicates that mKate2 is an appropriate acceptor for LSSmOrange for FRET-FLIM studies. By using a bi-exponential model, we were able to quantify this decay with a FRET lifetime of 1.18 ± 0.12 ns and a fraction of donor exhibiting FRET (f_D_) of 0.54 ± 0.02. These values are similar to other validated FRET pairs for FLIM such as Enhanced Green Fluorescent Protein (EGFP)/mCherry (f_D_ = 0.51) and mTFP1/EYFP (f_D_ = 0.73) with the same TCSPC method^31^.

Next, using the dual colour fastFLIM system combining the fastFLIM prototype^30^ and a dual view system (see Methods for details), we confirmed that LSSmOrange fluorescence emission was only recorded in the orange channel and not in the blue channel (Fig. 1B). In cells expressing the donor alone, we measured a mean fluorescence lifetime for LSSmOrange of 2.76 ± 0.03 ns (Fig. 1B). However, when expressed in tandem with mKate2 we measured a decreased mean lifetime of 2.32 ± 0.08 ns (Fig. 1B), corresponding to a mean FRET efficiency of 0.16 (calculated using: pseudoE = 1 − <τ>/<τ>_D_ where <τ>_D_ relates to the lifetime of the donor alone^31^). Again here, quantitative comparison of LSSmOrange/mKate2 pair with the two other FRET pairs characterized for FRET-FLIM indicated an intermediate FRET efficiency higher for the EGFP/mCherry FRET pair (mean FRET efficiency of 0.09), but still lower for the mTFP1/EYFP FRET pair (mean FRET efficiency of 0.23)^31^. mTFP1^32^ used in the aforementioned study is an appropriate cyan donor for FRET by FLIM^31^.

Combining single excitation wavelength at 440 nm with the cyan and orange channels of the dual colour FLIM set up, we measured mTFP1 and LSSmOrange fluorescence lifetimes expressed either alone or together in the same U2OS cells (Supplementary Figure S1). When expressed alone most of mTFP1 fluorescence emission was recovered in cyan channel. However, due to the large emission spectrum of mTFP1 (from 475 nm to 525 nm at half maximum), spectral bleed-through was detectable in the orange channel. It is noteworthy that mTFP1 fluorescence intensity levels were insufficient to determine a fluorescence lifetime in this channel. An average fluorescence lifetime of 2.62 ± 0.03 ns for mTFP1 was measured in the cyan channel. When LSSmOrange was expressed alone, as expected no signal was detectable in cyan channel and LSSmOrange average fluorescence lifetime measured in the orange channel was 2.76 ± 0.03 ns. When both fluorescent proteins were co-expressed in U2OS cells, average fluorescence lifetimes were 2.62 ± 0.03 ns for mTFP1 and 2.76 ± 0.04 ns for LSSmOrange, in the cyan and the orange channel respectively. In spite of the few photons coming from the mTFP1 spectral bleed-through in the orange channel, the LSSmOrange lifetime determination was unaffected. Our results indicate that these fluorescent proteins can be simultaneously used as donors in single wavelength excitation dual colour FLIM experiments.

### ShadowG is an efficient dim-fluorescent FRET acceptor for mTFP1 to reduce acceptor spectral bleed-through in LSSmOrange channel when using dual colour FLIM

Next, we co-expressed mTFP1-EYFP and LSSmOrange-mKate2 tandems in U2OS cell to determine whether both FRET acceptors, EYFP and mKate2, would be suitable with the single excitation wavelength dual colour FLIM approach (Supplementary Figure S2). Since the lifetime of a fluorophore is independent from the concentration, mTFP1 fluorescence lifetime was unaffected by the fluorescence intensity level and thus the amount of mTFP1 construct expressed in the cell. As expected, mTFP1 fluorescence lifetimes were unaffected by mTFP1-EYFP tandem expression levels (Supplementary Figure S2, cyan channel, lifetime images). Conversely, a striking increase of 350 ps in LSSmOrange fluorescence lifetime was calculated depending on mTFP1-EYFP fluorescence intensity (Supplementary Figure S2). While the mTFP1 spectral bleed-through did not affect the LSSmOrange fluorescence lifetime (Supplementary Figure S1), the spectral bleed-through of EYFP in the LSSmOrange donor channel contributed to LSSmOrange fluorescence lifetime heterogeneity.

To overcome this problem, we took advantage of dim-fluorescent (“dark”) acceptors recently developed for FRET analysis^26^,^33^. In FLIM, FRET is quantified by shortening of donor fluorescence lifetime, which requires an acceptor with a high molar absorption coefficient. However, for intramolecular FLIM-FRET measurements, the brightness of the acceptor is not relevant. Thus, we swapped the EYFP acceptor in our tandems with two different dim-acceptors: sREACh^33^, a EYFP mutant, excited at 514 nm or ShadowG^26^, a sREACh mutant with a blue shift spectrum. ShadowG, which is excited at 470 nm (same excitation spectrum as EGFP), is 114 fold dimmer than EYFP and 18 fold dimmer than sREACh. Comparing the fluorescence lifetime of mTFP1-EYFP, mTFP1-sREACh and mTFP1-ShadowG tandems with that of mTFP1 alone measured in U2OS cells indicated that sREACh and ShadowG are also reliable acceptors for mTFP1 (Supplementary Figure S3). This was further confirmed by mean FRET efficiency values of 0.23, 0.22 and 0.18, for mTFP1-EYFP, mTFP1-sREACh and mTFP1-ShadowG tandems, respectively. Even if little amounts of spectral bleed-through were detected for all conditions (Supplementary Figure S3: second row of fluorescence intensity images), mTFP1-ShadowG turned out to be a good FRET-FLIM pair to be combined with LSSmOrange/mKate2 (Supplementary Figure S3: second row of fluorescence lifetime images), as we found similar bleed-through levels comparable to mTFP1 alone (Supplementary Figures S1 and S3).

**Figure 2:**
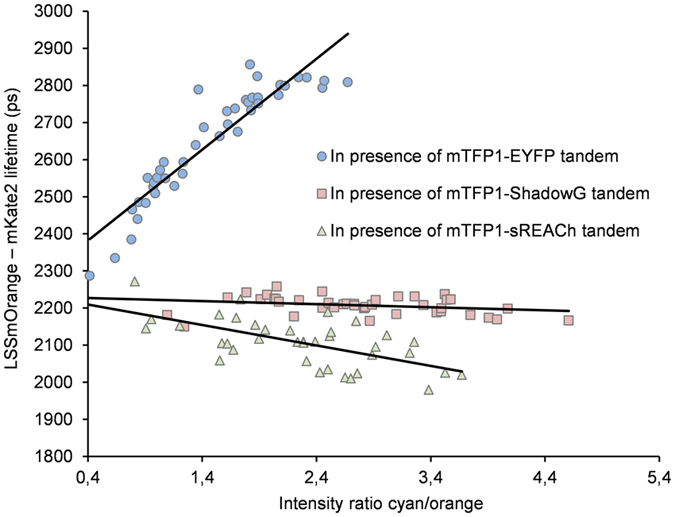
Using mTFP1-ShadowG FRET pair results in consistent LSSmOrange-mKate2 fluorescence lifetime determination by dual colour FLIM. Graphical representation of LSSmOrange-mKate2 lifetimes in the presence of mTFP1-EYFP, mTFP1-sREACh, or mTFP1-ShadowG plotted as a function of cyan/orange channel intensity ratio. U2OS cells were co-transfected with a fixed amount of LSSmOrange-mKate2 plasmid (0.5 μg/well) and increasing amounts of mTFP1-YFP, mTFP1-sREACh or mTFP1-ShadowG plasmids (0.25, 0.5, 075 or 1 μg/well). Each point corresponds to one cell. In the presence of mTFP1-EYFP (crosses, n = 42), LSSmOrange-mKate2 lifetime was markedly increased (τ_LSSmOrange-mKate2_ = 2,47 ns and 2,82 ns at intensity ratio of 0.4 up to 2.67, respectively). In presence of mTFP1-sREACh (triangle n = 47), LSSmOrange-mKate2 lifetime was decreased (τ_LSSmOrange-mKate2_ = 2,27 ns and 2,15 ns at intensity ratio of 0.81 up to 3.67, respectively). In the presence of mTFP1-ShadowG (squares n = 40), LSSmOrange-mKate2 lifetime remained stable (τ_LSSmOrange-mKate2_ = 2.21 ± 0.02 ns at intensity ratio of 1.09 up to 4.07).

To further investigate the bleed-through effect in our dual FLIM set up, LSSmOrange-mKate2 fluorescence lifetime and potential associated spectral bleed-through signals in the orange channel were measured in cells spanning mTFP1-Yellow fluorescence expression with blue to orange intensity ratio ranging from 0.4 to 4.4 (Fig. 2). In the presence of mTFP1-EYFP, LSSmOrange-mKate2 fluorescence lifetime increased from 2.47 ns to 2.82 ns (Fig. 2, crosses), representative images are shown in Supplementary Figure S2. A decrease in the average lifetime from 2.27 ns to 2.15 ns was observed in the presence of mTFP1-sREACh (Fig. 2, triangle). Albeit slight, LSSmOrange-mKate2 fluorescence lifetime did decrease for intensity ratio values spanning 0.81 up to 3.67 as represented by the linear regression. Even if spectral bleed-through of sREACh was very dim, the reported short sREACh fluorescence lifetime (τ_sREACh_ = 0.32 ns) could provide an explanation^33^ for this small decrease in lifetime. Finally, in presence of mTFP1-ShadowG (Fig. 2, squares), LSSmOrange-mKate2 fluorescence lifetimes measured in the orange channel were stable, 2.21 ± 0.02 ns (n = 40), across all intensity ratios tested ranging from 1.09 to 4.07. Here, we can conclude that mTFP1-Yellow spectral bleed-through contribution to LSSmOrange fluorescence lifetime in the orange channel is extremely low with ShadowG, as compared to EYFP or sREACh.

To summarize, we have shown that LSSmOrange exhibits mono-exponential fluorescence decay and that mKate2 is a reliable acceptor for LSSmOrange. In single excitation dual colour FLIM-FRET measurements, LSSmOrange fluorescence lifetime was not affected by mTFP1 spectral bleed-through in the orange channel. More importantly, the use of ShadowG as an acceptor for mTFP1 allows for consistent LSSmOrange fluorescence lifetime determination. Taken together, we validated the LSSmOrange/mKate2 and mTFP1/ShadowG FRET pairs as best candidates for our single excitation wavelength dual colour FLIM methodology towards multiplexing genetically encoded FRET biosensors.

### AKAR^dual^ and EKAR^dual^ validation for single excitation wavelength dual colour FLIM report on EGF-mediated PKA activation in HeLa cells

As a proof of feasibility, we applied our approach to the simultaneous monitoring of two kinases, PKA and ERK1&2. Two main reasons motivated our choice. From the biological point of view, the interplay between cAMP/PKA and MAPK/ERK1&2 signalling pathways has long been established (for review^11^). Both cascades modulate common cellular processes, and multiple levels of crosstalk depending on the cellular type and the cellular context have been described^10^,^11^,^15^,^34^,^35^. Because ERK1&2 is one of the cAMP targets associated with cell proliferation, emphasis has been put on hormone-mediated cAMP/PKA effect on ERK1&2 activation. So, until recently^35^, the Growth Factor (GF)-induced PKA activation effect has been less studied. From a biotechnological standpoint, we know from our own work^36^ and that of others^28^,^37^ that modifying FRET pairs has an impact on the performance of KARs. Fortunately, both AKAR^29^,^38^,^39^ and EKAR^28^,^37^,^40^,^41^ have already passed various rounds of optimization, which steadily increased their respective performance^28^,^29^. Accordingly, we anticipated that changing fluorescent proteins on biosensors would not have a major impact on the kinase activity of our biosensors.

**Figure 3:**
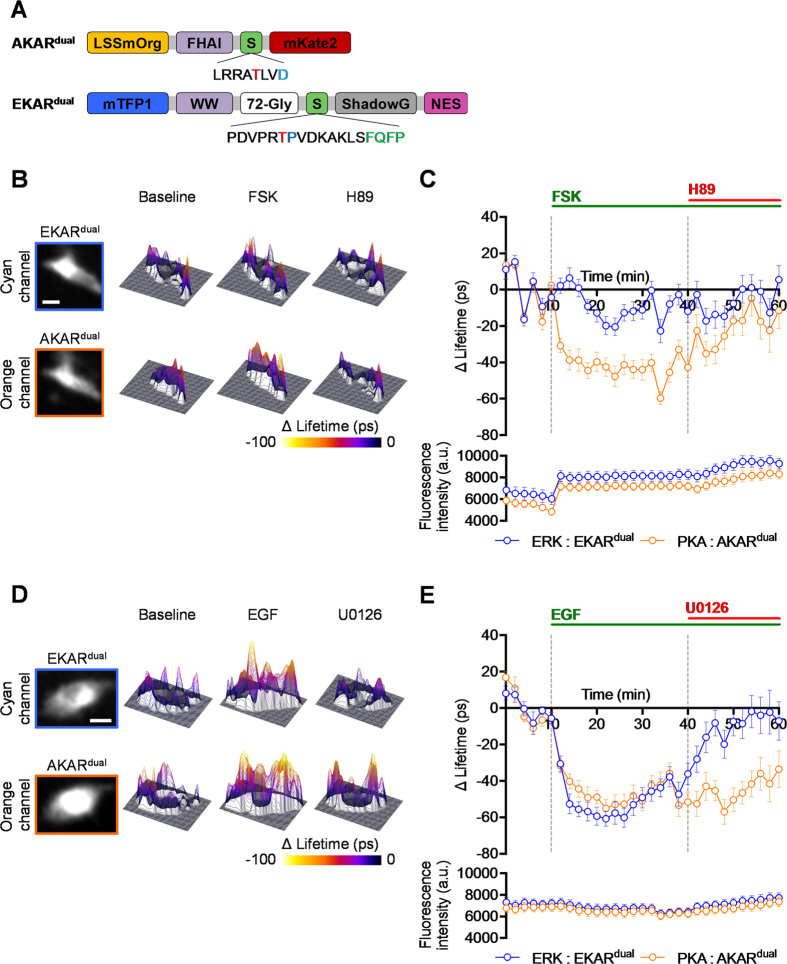
Validation of AKAR^dual^ and EKAR^dual^ using single wavelength excitation dual colour FLIM reveals an EGF-mediated PKA activation in HeLa cells. (**A**) Illustrations represent AKAR^dual^ (up) and EKAR^dual^ (down) biosensors. Biosensors were re-engineered with the following pairs of fluorescent protein: mTFP1/ShadowG for EKAR^dual^ and LSSmOrange/mKate2 for AKAR^dual^. Substrates of ERK1&2 or PKA kinases, specific recognition domains of the phosphorylated substrates, and the ERK1&2 docking site in EKAR^dual^ were unmodified and so kept identical to the respective original biosensors. For both sets of treatment, representative fluorescence intensity (most left images) and associated 3D ΔLifetime images are shown for each channel: cyan for EKAR^dual^ (top row) and orange for AKAR^dual^ (bottom row). (**B**) and (**D**) baseline (left column), activation by Fsk or EGF (middle column) and H89 or U0126 inhibition (right column), respectively. Graphs represent mean EKAR^dual^ and AKAR^dual^ ΔLifetime along time (60 min) during baseline (mean ΔLifetime from t = 0 min to t = 10 min of 0 ± 2 ps for both EKAR^dual^ and AKAR^dual^ in **C** and **E**), upon activation by Fsk or EGF (mean ΔLifetime from t = 12 min to t = 40 min of −9 ± 2 ps for EKAR^dual^ and −42 ± 1 ps for AKAR^dual^ in **C** and of −49 ± 2 ps for EKAR^dual^ and −46 ± 2 ps for AKAR^dual^ in **E**), and H89 or U0126 inhibition (mean ΔLifetime from t = 42 min to t = 60 min of −6 ± 3 ps for EKAR^dual^ and −20 ± 3 ps for AKAR^dual^ in **C** and of −10 ± 3 ps for EKAR^dual^ and −44 ± 3 ps for AKAR^dual^ in **E**) phases. Graphs, underneath mean EKAR^dual^ and AKAR^dual^ ΔLifetime graphs, represent fluorescence intensity along time (60 min) during baseline, upon activation by Fsk or EGF and H89 or U0126 inhibition phases. Fsk/H89 (n = 20), EGF/U0126 (n = 26). [Fsk] = 12.5 μM, [H89] = 20 μM, [EGF] = 100 ng/ml, and [U0126] = 20 μM. Fluorescence lifetime curves were calculated as a mean ± SEMs. (n) indicates the number of cells from at least 3 independent experiments. Scale bar = 10 μm.

We generated new versions of EKAR2G^28^ and AKAR4^29^, named EKAR^dual^ and AKAR^dual^ (Fig. 3A), that encode the FRET pairs mTFP1/ShadowG and LSSmOrange/mKate2, respectively. Standard biosensing reference experiments were carried out in HeLa cells expressing either AKAR^dual^ or EKAR^dual^ using the dual colour-FLIM system. To verify biosensor appropriate behaviour and sensitivity, we first recorded single kinase activity profiles upon dedicated activation and subsequent inhibition of either cAMP/PKA or ERK1/2 signalling pathways (Supplementary Figure S4). Forskolin/IBMX or EGF treatment resulted in a mean AKAR^dual^ and EKAR^dual^ fluorescence lifetime decrease characteristic of PKA and ERK1/2 pathway activation, which could be inhibited by either H89 or U0126, respectively. We could conclude from this first characterization step that AKAR^dual^ and EKAR^dual^ both exhibited the typical behaviour of the respective original biosensors^28^,^29^. To control the basal levels of activation for the two biosensors in our experimental condition, we have performed inhibitory treatments (U0126 and H89) for cells co-expressing AKAR^dual^ and EKAR^dual^ (Supplementary Figure S5). This supports the conclusion that the resting Hela cells show mild activation, as expected in an equilibrium in a biological system between kinases and phosphatases.

To confirm that the spectral bleed-through had no impact on PKA kinase activity measurements, we crosschecked ERK1&2 and PKA kinase activity profiles by applying dedicated biosensing reference experiments to each. HeLa cells transfected with both AKAR^dual^ and EKAR^dual^ were subjected to either forskolin/H89 or to EGF/U0126 treatment, and both PKA and ERK1&2 kinase activity profiles were recorded by single excitation wavelength dual colour FLIM (Fig. 3). We use a 3D representation for ΔLifetime images to enhance the visualization of kinase activity variations. As expected, the cAMP-mediated PKA activation induced by forskolin resulted in a decrease of AKAR^dual^ fluorescence lifetime (ΔLifetime of −40 ± 5 ps at t = 20 min), while having a non-significant decrease on ERK1&2 activity (ΔLifetime of −13 ± 5 ps at t = 20 min). H89-mediated PKA inhibition counteracted the recorded PKA activation by an increase in AKAR^dual^ fluorescence lifetime back to the basal activity level (ΔLifetime of −12 ± 10 ps at t = 60 min) (Fig. 3B and C). When monitoring ERK1&2 and PKA kinase activities upon EGF/U0126 treatment (Fig. 3D and E), EKAR^dual^ presented its characteristic behaviour. First a decrease and then an increase of EKAR^dual^ fluorescence lifetime were measured upon EGF (ΔLifetime of −59 ± 5 ps at t = 20 min) and subsequent U0126 treatment (ΔLifetime of −7 ± 11 ps at t = 60 min), respectively. Interestingly, an increase of PKA activity was also recorded in response to EGF, as witnessed by the marked decrease of fluorescence lifetime for AKAR^dual^ (ΔLifetime of −49 ± 4 ps at t = 20 min), which was not significantly affected by U0126 treatment (ΔLifetime of −34 ± 10 ps at t = 60 min). Note that the FLIM prototype exhibits a lifetime noise of ±20 ps (standard deviation). Any variation within the scale of this noise cannot be considered as a significant variation. For example the slight fluctuation in lifetime in Fig. 3E before EGF treatment cannot be attributed any meaningful biological relevance since it is within the measured noise range of the microscope system. We have simultaneously verified that no photobleaching occurs during lifetime acquisition by monitoring the intensity of the first time-gated image (Fig. 3C and E, bottom). Surprisingly, noise fluctuations are correlated for EKAR^dual^ (cyan channel) and for AKAR^dual^ (orange channel), for instance at t = 34 min in Fig. 3C. But this correlation does not come from spectral bleed-through as generally observed in conventional ratiometric measurements. Here we use the same excitation wavelength and the same time-gated image detection using the dual-view. Detection noise is then naturally correlated. Finally, to be convinced that the potential spectral bleed-through had no impact on our measurements, we performed experiments in which lifetime variation occurs for the cyan species while no variation will be expected in the orange channel. To do so, we have co-expressed EKAR^dual^ with LSSmOrange or the tandem LSSmOrange-mKate2 in Hela cells and we have performed ERK activation experiment (Supplementary Figure S6). We show that the variations of EKAR^dual^ lifetime consecutive of EGF activation and U0126 inhibition do not have any impact on LSSmOrange or LSSmOrange-mKate2 lifetimes in the orange channel, demonstrating that the potential contributions of cyan species in the orange channel are not detectable whatever the measured lifetime from 2.76 ns (LSSmOrange) to 2.32 ns (tandem LSSmOrange-mKate2).

**Figure 4:**
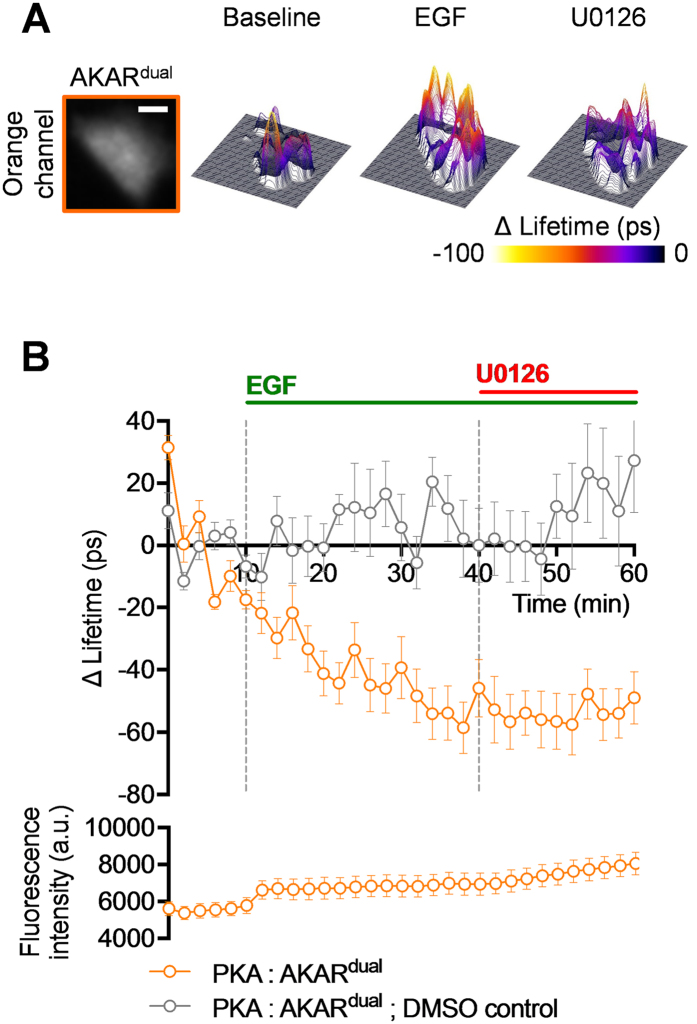
EGF-mediated PKA activation in HeLa cells confirmed on dual colour FLIM using AKAR^dual^. (**A**) Representative fluorescence intensity (most left image) and associated 3D ΔLifetime images are shown for the orange channel. AKAR^dual^ (n = 19): baseline (left), activation by EGF (middle) and U0126 inhibition (right). (**B**) The orange graph represents mean AKAR^dual^ ΔLifetime along time (60 min) during baseline (mean ΔLifetime from t = 0 min to t = 10 min of 0 ± 3 ps), upon activation by EGF (ΔLifetime of −41 ± 9 ps at t = 20 min), and U0126 inhibition (mean ΔLifetime from t = 42 min to t = 60 min of −53 ± 3 ps) phases. The grey graph represents DMSO control. Graph, below of mean AKAR^dual^ ΔLifetime graph, represents fluorescence intensity along time (60 min) during baseline, upon activation by EGF and U0126 inhibition phases. [DMSO] = 1/1000, [EGF] = 100 ng/ml, and [U0126] = 20 μM. Fluorescence lifetime curve was calculated as a mean ± SEMs. (n) indicates the number of cells from at least 3 independent experiments. Scale bar = 10 μm.

The previous section presents two interesting results in our cellular model: (i) FSK treatment does not activate ERK and (ii) EGF treatment activates PKA shown in an earlier report where an EGF-mediated PKA activity increases in mammalian cells^42^,^43^. First, using the new version of EKAR sensor (eCFP/YPet, unpublished optimized version) and ratiometric FRET measurements, we provided evidence that FSK does not activate ERK in Hela cells, confirmed by western blot using di-phospho ERK1&2 antibody (Supplementary Figure S7). Second, we performed two additional sets of experiments to confirm the EGF-mediated PKA activation in HeLa cells. In the former, a decrease of AKAR^dual^ mean fluorescence lifetime upon EGF treatment (ΔLifetime of −41 ± 9 ps at t = 20 min) symptomatic of an increase of PKA activity which was unaffected by U0126 treatment (mean ΔLifetime from t = 42 min to t = 60 min of −53 ± 3 ps) was observed in HeLa cells (Fig. 4). This decrease has to be compared to DMSO control where no significant change was observed in the same experimental regime (black curve). Note that at our excitation regime, no photobleaching was observed for AKAR^dual^ intensity (Fig. 4B bottom). In the latter, using the original AKAR4 (Cerulean/cpVenus) biosensor^29^ and ratiometric FRET measurements we confirmed the EGF-mediated PKA activation in HeLa cells (Supplementary Figure S8). One could ask how this EGF-mediated PKA activation might shape ERK1&2 kinase activity in HeLa cells.

### cAMP inhibition of EGF-mediated ERK1&2 activity is uncoupled from PKA activity level

**Figure 5:**
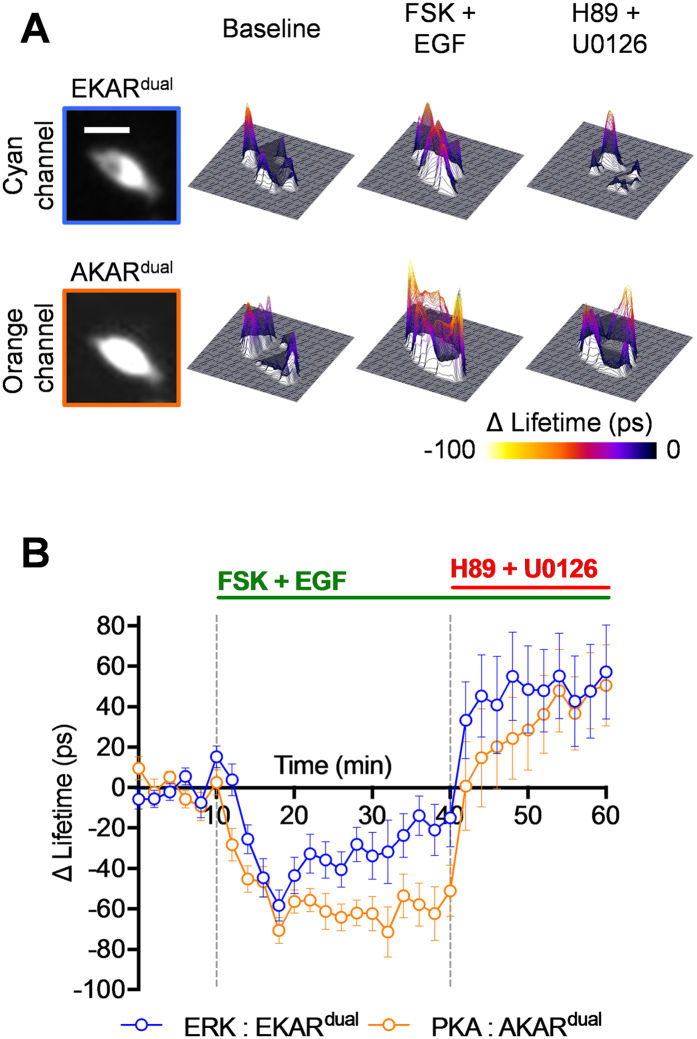
Forskolin treatment negatively impacts EGF-mediated ERK1&2 activation in HeLa cells. (**A**) Representative fluorescence intensity (most left images) and associated 3D ΔLifetime images are shown for each channel: cyan for EKAR^dual^ (top row) and orange for AKAR^dual^ (bottom row): Baseline (left column), co-activation by Fsk and EGF (middle column), and H89 and U0126 inhibition (right column), respectively. (**B**) Graph represents mean EKAR^dual^ (blue) and AKAR^dual^ (orange) ΔLifetime along time (60 min) during baseline (mean ΔLifetime from t = 0 min to t = 10 min of 0 ± 3 ps for EKAR^dual^ and 0 ± 3 ps for AKAR^dual^), upon co-activation by Fsk and EGF (peaking at ΔLifetime of −58 ± 7 ps for EKAR^dual^ and −70 ± 6 ps for AKAR^dual^ at t = 18 min), and H89 and U0126 inhibition (ΔLifetime of + 45 ± 20 ps for EKAR^dual^ and + 15 ± 24 ps for AKAR^dual^ at t = 44 min) phases; n = 26. [Fsk] = 12.5 μM, [H89] = 20 μM, [EGF] = 100 ng/ml, and [U0126] = 20 μM. Fluorescence lifetime curves were calculated as a mean ± SEMs. (n) indicates the number of cells from at least 3 independent experiments. Scale bar = 10 μm.

Taking into consideration that different modes of cAMP and PKA activation can lead to a contradictory effect on ERK1&2, we finalized our study by exploring the effect of cAMP- versus EGF-induced PKA activation on ERK1&2 activity. Using HeLa cells co-expressing AKAR^dual^ and EKAR^dual^, we simultaneously monitored PKA and ERK1/2 activities by single excitation wavelength dual colour FLIM, upon co-activation and co-inhibition of both signalling pathways (Fig. 5). EGF-mediated ERK1&2 and forskolin-mediated-cAMP-dependent PKA activations resulted in an almost concomitant activation of both PKA and ERK1&2, peaking around 8 minutes after stimulation (ΔLifetime of −58 ± 8 ps for EKAR^dual^ and −71 ± 6 ps for AKAR^dual^ at t = 18 min). However, the ERK1&2 activation profile upon combined forskolin and EGF stimulations was somewhat different from that of EGF treatment alone (Fig. 3B and Supplementary Figure S4B). Indeed, it resulted in a more transient ERK1&2 activation (ΔLifetime of −34 ± 12 ps at t = 30 min), while PKA activity remained relatively stable until H89 inhibitor treatment (ΔLifetime of −62 ± 9 ps at t = 30 min), as previously shown (Fig. 3A and Supplementary Figure S4A). While dual pathway inhibitions produced the symptomatic decrease in both PKA and ERK1&2 kinase activities, we note that in these experimental conditions the inhibition reverted both kinase activity levels beyond baseline levels (ΔLifetime of 51 ± 20 ps for AKAR^dual^ and ΔLifetime of 57 ± 23 ps for EKAR^dual^ at t = 60 min). We show a cAMP-mediated reversal on EGF-mediated ERK1&2 activity increase. Compelling evidences demonstrated that the ERK1&2 cascade is regulated by cAMP/PKA pathway (for review^10^). This crosstalk was reported to modulate the duration and the strength of ERK1/2 activity^35^, mainly in a Raf-isoform-dependent manner^11^.

**Figure 6:**
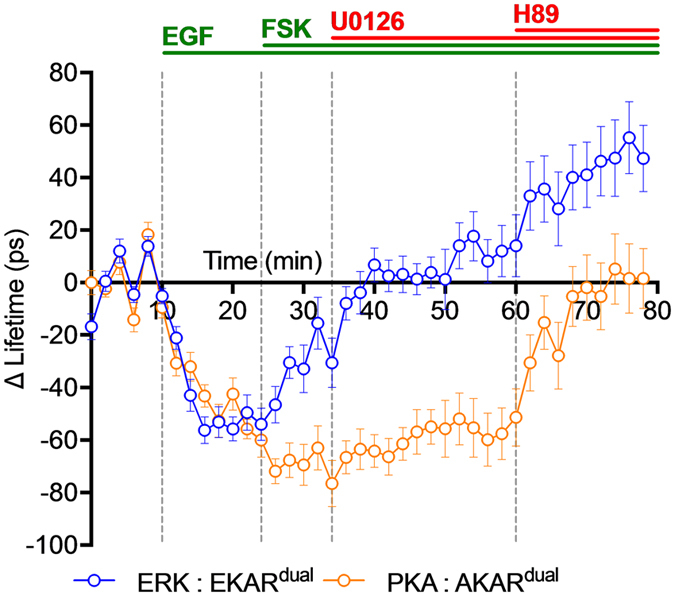
Increasing cAMP levels reverts EGF-mediated ERK1&2 activation while reinforcing PKA activity. ERK1&2 and PKA kinase activity profiles measured in HeLa cells co-expressing EKAR^dual^ and AKAR^dual^ treated by indicated activators and inhibitors, using single wavelength excitation dual colour FLIM system. Graph represents mean EKAR^dual^ (blue) and AKAR^dual^) ΔLifetime along time (78 min) during baseline (ΔLifetime of 0 ± 2 ps for EKAR^dual^ and 0 ± 2 ps for AKAR^dual^ over 10 min), upon activation EGF (peaking of ΔLifetime of −56 ± 5 ps for EKAR^dual^ and −43 ± 4 ps for AKAR^dual^ at t = 16 min) followed by Fsk/IBMX treatment (ΔLifetime of −33 ± 9 ps for EKAR^dual^ and −69 ± 8 ps for AKAR^dual^ at t = 30 min) and U0126 (ΔLifetime of −4 ± 8 ps for EKAR^dual^ and −63 ± 8 ps for AKAR^dual^ from inhibition to t = 38 min) followed by H89 inhibition phases (ΔLifetime of 35 ± 13 ps for EKAR^dual^ and −15 ± 10 ps for AKAR^dual^ at t = 64 min); n = 19. [Fsk] = 12.5 μM, [H89] = 20 μM, [EGF] = 100 ng/ml, and [U0126] = 20 μM. Fluorescence lifetime curves were calculated as a mean ± SEMs. (n) indicates the number of cells from at least 3 independent experiments.

To overcome problems with competition or synergistic effects with activator and inhibitors, we proceeded with a different treatment protocol. EGF and then Forskolin/IBMX stimulations, followed by U0126 and then H89 inhibitions were successively applied (Fig. 6). EGF stimulation resulted in concomitant ERK1&2 and PKA activations (ΔLifetime of −43 ± 4 ps for AKAR^dual^ and −56 ± 5 ps for EKAR^dual^ at t = 16 min) as previously measured. The subsequent Forskolin/IBMX treatment potentiated PKA activation (ΔLifetime of −69 ± 8 ps for AKAR^dual^ at t = 30 min) while reverting ERK activation (ΔLifetime of 33 ± 9 ps for EKAR^dual^ at t = 30 min) in a way comparable to what was initially observed with the combined EGF/Forskolin stimulation (Fig. 5). Upon U0126 treatment, ERK1&2 activity returned to basal level (ΔLifetime of −4 ± 8 ps at t = 38 min), without affecting PKA activity (ΔLifetime of −63 ± 8 ps at t = 38 min). Only addition of H89, precipitated PKA activity back to basal level (ΔLifetime of −15 ± 10 ps at t = 64 min) and further decreased ERK1&2 activity levels (ΔLifetime of 36 ± 13 ps at t = 64 min) due to H89 off target effects. This came as no surprise due to the known multifaceted pharmacology of H89, which has also been reported to inhibit other kinases including the mitogen activated protein kinase kinase-1 (MEK1)^44^. The simultaneous recording of PKA and ERK1&2 kinase activities revealed concomitant EGF-mediated activations of both kinases in HeLa cells. In this situation the subsequent Forskolin-induced cAMP release reversed the transient increase of EGF-mediated ERK1&2 kinase activity while reinforcing PKA activation.

This last set of experiments confirmed that we were able to monitor two kinases activity profiles modulated by different stimulus, with no lag time or spatial distribution discrimination. Importantly, if the spectral bleed-through had been detrimental, we would never have been able to measure such independent variations of PKA and ERK1&2 signalling pathways. Overall, we confirm that our methodology is well adapted for multiplexing of genetically encoded FRET biosensor.

## Discussion

Here we report on the validation of a multiplexed approach to follow two genetically encoded FRET-based kinase activity reporters at the same time in the same sample and in the same cellular compartment. Commonly, FRET is measured by the fluorescence intensity ratio of the acceptor to the donor. In that case, whatever the two fluorescent protein FRET pairs chosen, CFP/YFP and mOrange/mCherry^21^, mTFP1/mCitrine and mAmetrine/tdTomato^22^,^23^, mTagBFP/sfGFP and mVenus/mKok^24^, the multiplexed approach suffers from two limitations: (i) a spectral bleed-through of the first acceptor in the second donor emission band that depends directly on the respective quantities of the two biosensors and (ii) the multiple excitation wavelength which requires sequential acquisition, and thus is not adequate to follow fast signal dynamics or signal changes in highly motile sample.

To overcome the first limitation, a meroCBD biosensor modified with a far red organic fluorophore (Alexa750) was used for probing Cdc42 simultaneously with a genetically encoded CFP/YFP FRET-based biosensor for Rho A^45^. This approach prevents spectral bleed-through but cannot be generalized to all genetically encoded FRET biosensors where organic fluorophores are not easily utilised to replace fluorescent proteins. Recently, an elegant method based on linear unmixing of 3D excitation/emission fingerprints applied to three biosensors simultaneously was published^46^. This type of approach based on image calculation is often limited by the different biosensors expression level and a poor signal to noise ratio after complex image corrections. In our approach, we use FLIM instead of ratio imaging to measure FRET. When FRET occurs, donor fluorescence lifetime decreases. The method requires measurement of the donor fluorescence only and is independent of emission from the acceptor. Thus we can use a dim-fluorescent acceptor such as ShadowG to overcome the problems of spectral bleed-through. Other multiplex studies were carried out utilizing FLIM. By using CFP and YFP as donor and the same red acceptor (tHcRed), FLIM of CFP and YFP donor allows distinguishing the two different FRET signals^47^. Combination of FLIM-FRET of a red-shifted TagRFP/mPlum pair with ratio imaging of a CFP/Venus pair allows maximal spectral separation while at the same time overcoming the low quantum yield of the far-red acceptor mPlum^48^. Actually, in these two last examples the spectral bleed-through was alleviated but not the limitation associated with the multiple excitations.

To overcome the second limitation, the two FRET pairs CFP/YFP and Sapphire/RFP in combination with a single violet excitation were used^49^ resulting in no lag time in biochemical activity recording. However, the spectral bleed-through and excitation crosstalk once again necessitates linear unmixing. Another interesting approach for multiplexing two FRET activities was developed by Schervakova and co-workers, where a large stokes shift orange fluorescent protein (LSSmOrange) was used. The authors combined a CFP-YFP together with LSSmOrange-mKate2 biosensors enabling imaging of apoptotic activity and calcium fluctuations in real time using intensity-based methods^25^. Note that in this work, apoptotic activity was monitored by cleaved FRET biosensor giving a 0–1 answer different to conformational FRET biosensor where quantification is required. In our method, we also take advantage of a CFP like protein together with LSSmOrange to be able to use a single 440 nm excitation. Moreover, because we combine this with dual colour FLIM approach, we are also able to significantly reduce the EYFP like spectral bleed-through in LSSmOrange channel by using a dim-fluorescent acceptor such as ShadowG. Our approach overcomes both limitations in the multiplexing experiments using one excitation wavelength for two FRET fluorescent protein pairs, and reducing the spectral bleed-through between biosensors by using ShadowG as an acceptor for mTFP1.

In a second stage, we wanted to assess whether our methodology would be a reliable one towards multiparametric biosensing. Because the study of the intricate relationship between cAMP/PKA and MAPK/ERK1&2 signal transduction pathways regularly provides new mechanistic insights^15^,^18^,^35^, we opted for it as a structured and defined playground for application purposes.

Our results using EKAR^dual^ and AKAR^dual^ clearly reflect the complexity of cAMP/PKA-MAPK/ERK1&2 interconnections. This crosstalk is usually presented from the following perspective: how does cAMP/PKA modulate MAPK/ERK1&2 activity^11^? Aside from hormone receptors signalling effects on PKA, GF-induced PKA activation was also long reported^50^, with a keen interest for PKA implication in tumour biology^51^.

Our first observation, using single excitation wavelength dual colour FLIM with specifically optimized PKA and ERK1&2 kinase activity reporters, is an EGF-mediated PKA activation concomitant to that of ERK1&2 in the cervical cancer HeLa cell line. The reported PKA-RI subunit preponderance in cancer cells and its direct interaction with the EGFR via the Gbr2 adaptor protein^51^, together with recent evidences of PKA-Cα subunit phosphorylation by RTKs^42^ can explain our observation. In addition, scaffolds proteins such as AKAP-Lbc and KSR-1, that were reported to favour discrete localizations of both PKA and ERK1&2 generating pockets of concentrated enzyme activities, were shown to provide platforms for mitogenic signal amplification^15^. Actually, compartmentalized GF-induced PKA activation modulating the features of ERK1&2 kinase activity was recently illustrated in an elegant study from Zhang’s group^35^. While monitoring of compartmentalized PKA and ERK1&2 enzyme activities was beyond the scope of our study, it could now be performed in a multiplex manner thus maximizing crosstalk relevance at the single living cell level.

Raf isoforms expression levels were shown to be implicated in the various responses exerted by cAMP/PKA on MAPK/ERK1&2 signalling^52^. Differences in ERK1&2 kinase activity mainly result from the differential activation of either Ras/C-Raf-1/MEK/ERK or Rap1/B-Raf/MEK/ERK^11^. Activation of the exchange protein directly activated by cAMP (Epac) upon cAMP increasing levels triggers activation of Rap1 and sustained activation of B-Raf/MEK/ERK pathway^53^. By contrast, cAMP-mediated PKA activation is generally known to directly inhibit C-Raf-1, mediated by AKAP preventing over-activation of cAMP-mediated ERK1&2 pathway. Our second observation would be in line with the latter since forskolin-induced cAMP levels increase reverted activation of EGF-mediated ERK1&2 while reinforcing PKA activity. However, cAMP was reported to either inhibit C-Raf-1 or activate B-Raf. Therefore, cells expressing low B-Raf level will preferentially impair mitogenic signals in response to cAMP release. Actually, B-Raf kinase activity level has been found to be low in HeLa cells relative to A-Raf, while being intermediate for C-Raf-1^54^. Thus, in Hela cells, MEK/ERK1&2 signalling upon a mitogenic stimulation is mainly mediated by A-Raf. Actually, A-Raf triggers a MEK-1 dependent ERK1&2 activation upon EGF-treatment^55^ with a known ERK1&2 nuclear translocation. Using the recently published ERK2-LOC translocation reporter^56^ and a similar ERK1-LOC would be an elegant way to control whether the observed EGF-mediated ERK1&2 activation is A-Raf/MEK1 dependent. Although cAMP/PKA-mediated A-Raf regulation remains unclear, it is generally agreed that the target of cAMP is C-Raf-1^2^. How and if PKA activity can contribute to both EGF-mediated ERK1&2 activation and Forskolin-induced reversal of EGF-mediated ERK1&2 kinase inhibition remains unanswered in this cellular context. Other possible explanations arise from recent studies involving a PDE isoforms (PDE8A), which associates with C-Raf to protect it from inhibitory phosphorylation by PKA^18^. This actually enhances C-Raf to stimulate ERK1&2 cascade. Another connection with MAPK/ERK1&2 at the PDEs level should be mentioned. Indeed, ERK2 was reported to phosphorylate the C-terminal catalytic domain of several PDEs^17^. Taken together one might speculate whether EGF- and cAMP-mediated signals exert different modus of active PKA.

Our methodology provides evidence of the relevance of a multidimensional kinase activity profiling in living cells. Indeed, we have depicted here a process where simultaneously ERK1&2 and PKA kinases activities recordings in HeLa cells were successfully carried out. Results obtained at the single living cell level, within the same cell, and at the same time provide a glimpse of how cells integrate in real-time external signals upon dual signalling pathway engagement. In this study, we have used FRET biosensor diffusing all over the cell. The sub-cellular localization of the activation is not easy to measure since the diffusion of the probe is too fast compared to the speed of measurement. A promising solution would be to localize the kinase biosensors with targeting signal peptides^29^. Questions raised from such multidimensional biochemical activities profiling pave the way for future experiments aiming at untangling cellular signalling crosstalk.

As a perspective, since FLIM is adequate for quantitative intermolecular FRET^57^, our approach is easily transposable to study protein-protein interactions. Additionally, the possibility to expand our method to three biosensors could be achieved by using a non-fluorescent acceptor for LSSmOrange and a third large stoke shift donor such as LSSmKate^58^ in combination with an infra-red acceptor such as IRFP670^59^.

## Methods

### Reagents

Human epithelial growth factor (EGF, #E9644), MEK inhibitor (U0126, #U120) and dimethylsulfoxide (DMSO, #D8418) were purchased from Sigma Aldrich. Forskoline (Fsk, #1099), an adenylate cyclase activator responsible for cAMP production, PKA inhibitor (H89 dihydrochloride, #2910) and phosphodiesterase inhibitor (IBMX, #2845) were purchased from Tocris.

### Plasmids

GFP-sREACh (mGFP-10-sREACh-N3 from Dr Ryohei Yasuda, Addgene plasmid #21947) and mTFP1 vectors were digested with *NheI* and *BamHI*. mTFP1 fragment was inserted into the corresponding restriction sites in the GFP-sREACh (−/sREACh) vector to create the new vector mTFP-sREACh. EGFP-Shadow (was gift from Dr Murakoshi, National Institute for Physiological Sciences, Japan) and mTFP1-sREACh vectors were digested with *BamHI* and *NotI*. ShadowG fragment was inserted into the corresponding restriction sites in the mTF1-sREACh (mTFP1/-) vector to create the new vector mTFP1-ShadowG. LSSmOrange (pLSSmOrange-C1 from Dr Vladislav Verkhusha, Addgene #37130 plasmid) was inserted in EGFP-C3 vector by digestion with AgeI and BsrGI to create a new vector LSSmOrange-C3. LSSmOrange-C3 generated is used to insert LSSmOrange in mKate2-N plasmid (from Dr Sergi Padilla-Parra) by digestion with NdeI and BamHI to create a new vector LSSmOrange-mKate2. FRET biosensor plasmid EKAR2G (monomeric Teal Fluorescent Protein (mTFP1)/Venus, Addgene plasmid #39813) was a gift from Dr. Olivier Pertz (Department of Biomedicine, University of Basel, Switzerland). AKAR4 plasmid was kindly provided by Dr. Jin Zhang (Department of Pharmacology and Molecular Sciences, Johns Hopkins University School of Medicine, Baltimore, USA). EKAR2G (mTFP1/Venus) vector was digested with *Not*I and *Kpn*I to release the Venus insert generating the EKAR2G (mTFP1/-) vector. ShadowG plasmid was a gift from Dr Murakoshi (National Institute for Physiological Sciences, Japan). ShadowG coding sequence was amplified from the ShadowG plasmid using the forward primer ShadowG.NotI (*Not*I site underlined) and the reverse primer ShadowG.KpnI (*Kpn*I site underlined). ShadowG coding sequence was then inserted into the corresponding restriction sites in the EKAR2G (mTFP1/-) backbone in frame with EKAR2G Molecular Recognition Element (MRE) and the upstream mTFP1 fluorescent protein to create the new expression vector EKAR^dual^ (mTFP1/ShadowG). AKAR4 (Cerulean/cpVenus) vector was digested to remove sequentially Cerulean (*BamH*I and *Sph*I) and cpVenus (*Sac*I and *EcoR*I) inserts. LSSmOrange and mKate2 fluorescent proteins were then amplified from pLSSmOrange-C1 (from Dr Vladislav Verkhusha, Addgene plasmid #37131) and pmKate2-N1 (from Dr Sergi Padilla-Parra) respectively using the following primer pairs (forward/reverse): LSSmOrange.BamHI-F/ LSSmOrange.SphI-R and LSS-mKate2.SacI-F/mKate2.EcoRI-R. LSSmOrange and mKate2 PCR products were subsequently sub-cloned into the corresponding restriction sites in the AKAR4 (−/cpVenus) and AKAR4 (LSSmOrange/-) backbones respectively in frame with the AKAR4 MRE to create the new expression vector AKAR4^dual^ (LSSmOrange/mKate2). All resulting constructs were verified by restriction digestion onto agarose gel electrophoresis and then validated by sequencing (VIB for AKAR4 derivatives and IGDR for EKAR2G derivatives). All enzymes and buffers were purchased from New England Biolabs. Oligonucleotides were synthesized by Integrated DNA Technologies (Belgium) and Sigma-Aldrich (France) AKAR4 and EKAR2G, respectively. DNA fragments were all purified on Qiagen plasmid purification columns (#28106, #28706 and #27106, Qiagen).

### Cell Culture and Transfection

HeLa cells were purchased from the European Collection of Cell Cultures (UK) and U2OS cells were a gift from Dr Gyula Timinszky (LMU, Munchen, Germany). HeLa and U2OS cells were maintained at 37 °C under 5% CO_2_ in Dulbecco’s Modified Eagle Medium (DMEM, #E15-009, PAA) supplemented with 10% fetal bovine serum (FBS, #A15-101, PAA), 1% penicillin/streptomycin (P/S, #15140-122, Gibco, Life Technologies) and 1% L-Glutamine (Glu, #25030024, Gibco, Life Technologies). For live cell imaging, cells were seeded on Lab-Tek 4 wells (#055078, Dominique Dutscher) at 60% confluence 24 h before transfection on day 1. On day 2, transfections were carried out using JetPrime reagent (#114-15, Polyplus) according to the manufacturer’s instructions. After 4 h of incubation with the transfection mixture, medium was replaced by 400 μl per well of culture medium composed of FluoroBrite phenol red-free medium (#A18967-01, Life Technologies) containing 0.1% FBS for overnight starvation. For experiment relating to Fig. 2, U2OS cells were transfected with 0.5 μg/well of LSSmOragne-mKate2 plasmid, and 0.25, 0.5, 0.75, and 1 μg/well of the different mTFP1-Yellow plasmids, in order to generate cells with an array of tandem constructs expression levels.

### Imaging for biosensing experiments

For cell treatments, activators or inhibitors were diluted in pre-heated FluoroBrite medium in a final volume of 100 μl and were carefully added to the well. Concentration used: [DMSO] = 1/1000, [Fsk] = 12.5 μM, [IBMX] = 75 μM, [H89] = 20 μM, [EGF] = 100 ng/ml, and [U0126] = 20 μM. Standard protocol for biosensing experiment was as follows. Cells were positioned on the microscope stage 30 min before the beginning of each experiment. The first 10 minutes of imaging where cells were untreated provided basal kinases activity levels (correspond to baseline phase). Upon signalling pathways activation, images were acquired for a 30 minutes period, which was prolonged of 20 minutes upon signalling inhibitor. Images were acquired at 2 minute intervals.

### FRET imaging

#### FLIM-FRET experiments

For the characterization of LSSmOrange fluorescence lifetime, we used a time correlated single photon counting (TCSPC) system. This system, a confocal microscope Leica SP8 (Manheim, Germany), is equipped with single molecule detection (SMD) module based on Picoquant hardware (Berlin, Germany). Briefly, a 440 nm diode pulsed laser at 40 MHz repetition rate is fiber-coupled to the confocal head of an inverted microscope with a 63× or 40× oil immersion objective (NA = 1.4 or 1.30 respectively) and the fluorescence emission after passing through pinhole set at 1 Airy unit is directed to the external port connected to a two colour coupling module with a dichroic mirror of 505 nm splits the fluorescence in two channels with 480/30 nm and 579/34 nm band pass filters. Two optical fibres are used to couple two single-photon avalanche diode (SPAD) detectors; photon counting detector (MPD from PDM) module for the cyan channel and a red sensitive SPAD (TauSPAD from Picoquant) for the orange channel used as Single Photon Counting detectors. A Picoharp 300 is used for Time-Correlation and for image reconstruction using scanning signals to recover FLIM images (time-tagged time resolved (TTTR) method). Lifetime value from cell to cell was determined by fitting the fluorescence decay of the whole cell Region Of Interest with a single exponential model.

For single excitation wavelength dual colour FLIM system we used our FastFLIM system. This FLIM system equipped with a pulsed white light laser tuned at 80 Mhz coupled to a live cell fluorescence microscope and dual colour emission coupled to a FLIM detection previously described in ref. 30. After wavelength selection of the supercontinuum at 440 nm (with about 10 nm narrow band of excitation), the laser is coupled to a multifocal spinning disk CSUX1 (Yokogawa, Japan) and cells were imaged using a 63× or 20× oil immersion objective (NA = 1.4 or 0.70 respectively). At the emission side, a Dual View (DV2 multichannel imaging system, Photometrics, USA) was used to split the field of view of the camera in two colours, using a dichroic mirror at 505 nm splitting the fluorescence emission in two channels: 480/30 nm and 579/34 nm band pass filters. Then the fluorescence image was directed to a High Rate Intensifier (Picostar, LaVision, Germany) coupled to a charged coupled device (CCD) camera (CoolSNAP HQ2, Photometrics, USA). Five time-gated images of 2 ns gate width were sequentially acquired (using 100 to 300 ms exposure time depending on the intensity of the sample) with different delays relative to the pulsed laser (from 0 to 8 ns). The time-gated stack of 5 images was then used for direct calculation of mean fluorescence lifetime in a pixel by pixel basis^60^.

#### Ratiometric FRET experiments

For sensitized emission measurements of AKAR4 biosensor in HeLa cells wide-field images were captured with a Nikon TiE inverted microscope with a 20 × 0.5NA objective and a DS-Qi2 CMOS camera (Nikon, Japan). Images were acquired at intervals of 2 min with the Nikon NIS-Elements acquisition software using JOBS module (Nikon, Japan). A Lumencor Spectra X LED Light Engine (Lumencor, Beaverton, OR, USA) was used as excitation light source to reduce phototoxicity. Ratio imaging used a 440/30 nm excitation filter, a t440/510/575rpc multi-band dichroic mirror, and two emission filters (ET480/40 M (cyan fluorescent protein: CFP) and AT545/30 M (FRET)). Lumencor provided excitation filters, and all dichroic mirrors and emission filters were obtained from Chroma Technology (Brattleboro, VT, USA). An automated emission filter wheel Lambda 10-B Smart Shutter (Sutter Instrument, Novato, CA, USA) was used. The F480/F545 emission ratio, indicative of biosensor activation, calculated for each pixel on the whole image, was performed with custom routines written in IGOR Pro environment (Wavemetrics, Lake Oswego, OR, USA). Normalized F480/F545 emission ratio values were then plotted in PrismV (GraphPad software, La Jolla, CA, USA) and displayed in two ways: cell trace for each individual cell (grey) overlaid with the population mean in red and the 25^th^ and 75^th^ percentile values in blue.

### Data analysis

For the TCSPC analysis, Symphotime software (Picoquant, Berlin, Germany) was used to analyze our imaging data. The channel 1 (cyan channel) or 2 (orange channel) was selected. Regions of Interest on different cells were fitted using a single or a double-exponential decay.

The fastFLIM system produces an intensity image from the first time-gated image and a lifetime image from the calculation of the direct first order temporal mean of the recorded decay (<τ> = Σt_i_ I_i_/Σ I_i_ where t_i_ and I_i_ correspond to the correlated time and the intensity of the time-gated channel I, respectively). Manual segmentation of the different cells was carried out to recover the mean lifetime at different time points of the experiments.

## Additional Information

**How to cite this article:** Demeautis, C. *et al*. Multiplexing PKA and ERK1&2 kinases FRET biosensors in living cells using single excitation wavelength dual colour FLIM. *Sci. Rep.* **7**, 41026; doi: 10.1038/srep41026 (2017).

**Publisher's note:** Springer Nature remains neutral with regard to jurisdictional claims in published maps and institutional affiliations.

## Supplementary Material

Supplementary Material
