# Supplementary material for: Multiplexing PKA and ERK1&2 kinases FRET biosensors in living cells using single excitation wavelength dual colour FLIM
Source: Sci Rep. 2017 Jan 20;7:41026. doi: 10.1038/srep41026 (PMC5247693; doi:10.1038/srep41026)
Supplement: Supplementary Material [file srep41026-s1.pdf]

Supplementary Material:

Multiplexing PKA and ERK1&2 kinases FRET biosensors in living cells using single excitation wavelength dual color FLIM.

Claire Déméautis, François Sipieter, Julien Roul, Catherine Chapuis, Sergi Padilla-Parra, Franck B. Riquet and Marc Tramier

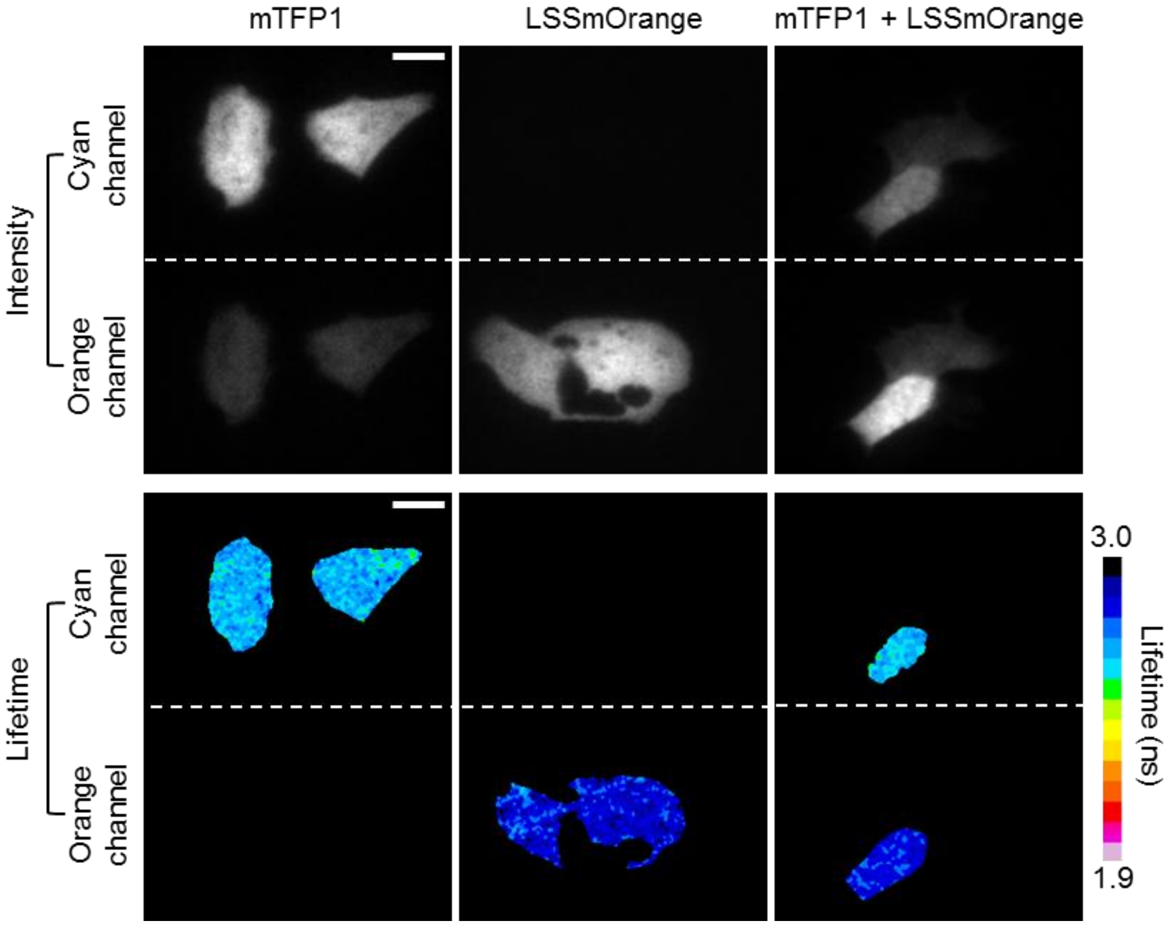

<u>Supplementary Figure 1: mTFP1 and LSSmOrange can be measured simultaneously by single wavelength excitation dual colour FLIM.</u>

Representative intensity and FLIM images acquired simultaneously in the cyan (480/30 nm) and in the orange (579/34 nm) channels in U2OS cells expressing either mTFP1 (left column) or LSSmOrange, (middle column) or both (right column) using a 440nm single wavelength excitation and dual color FLIM system. mTFP1 and LSSmOrange fluorescence lifetimes were recovered separately in the cyan and the orange channel, respectively. Due to the large emission spectrum of mTFP1, spectral bleed-through can be seen in the orange channel. Note that mTFP1 intensity levels were not sufficient for mTFP1 fluorescence lifetime determination in the orange channel. Average fluorescence lifetimes: Alone, τ_{mTFP1}=2.62±0.03ns (n=28), $\tau_{LSSmOrange}$=2.76±0.03ns (n=34); Co-expressed, τ_{mTFP1}=2.62±0.03ns (n=23), $\tau_{LSSmOrange}$=2.76±0.04ns (n=23). Fluorescence lifetimes were calculated as a mean±SD. (n) indicates number of cells from at least 3 independent experiments. Scale bar = 10 µm.

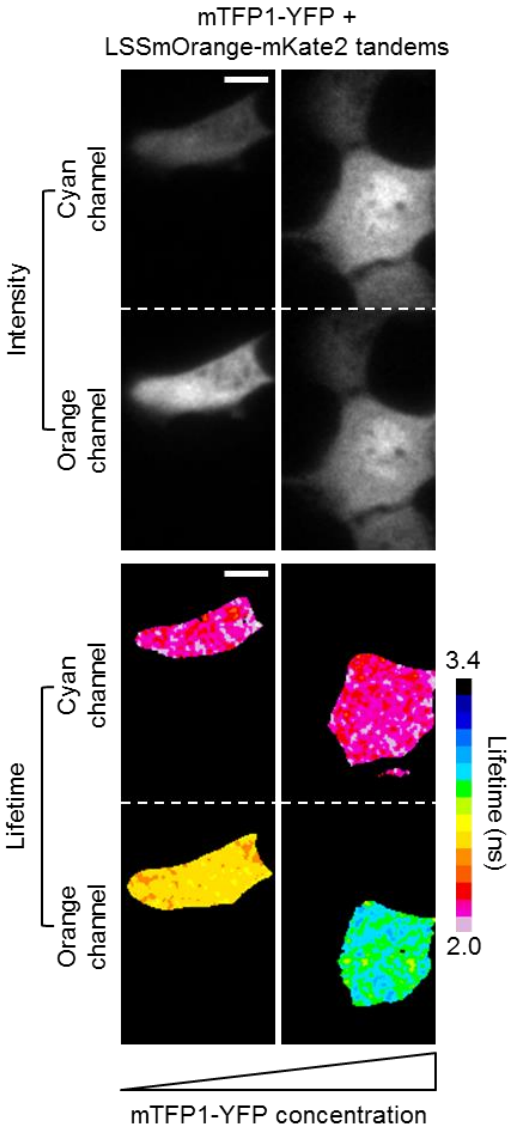

<u>Supplementary Figure 2: EYFP spectral bleed-through contributes to LSSmOrange-mKate2 fluorescence lifetime heterogeneity.</u>

Representative fluorescence intensity and lifetime images acquired using the dual color FLIM system in U2OS cells expressing mTFP1-YFP and LSSmOrange-mKate2. From left to right: intensity ratios are I_{cyan}/I_{orange} = 0.35 (left) and 1.25 (right); mean lifetimes are τ_{cyan}=2,02ns for both and τ_{orange}=2,47ns (left) and 2,82ns (right). Note the difference in mTFP1-EYFP fluorescence intensities levels in the cyan channel in presence of LSSmOrannge-mKate2 correlated with the measurement of a higher LSSmOrange fluorescence lifetime in the orange channel. Scale bar = 10 µm.

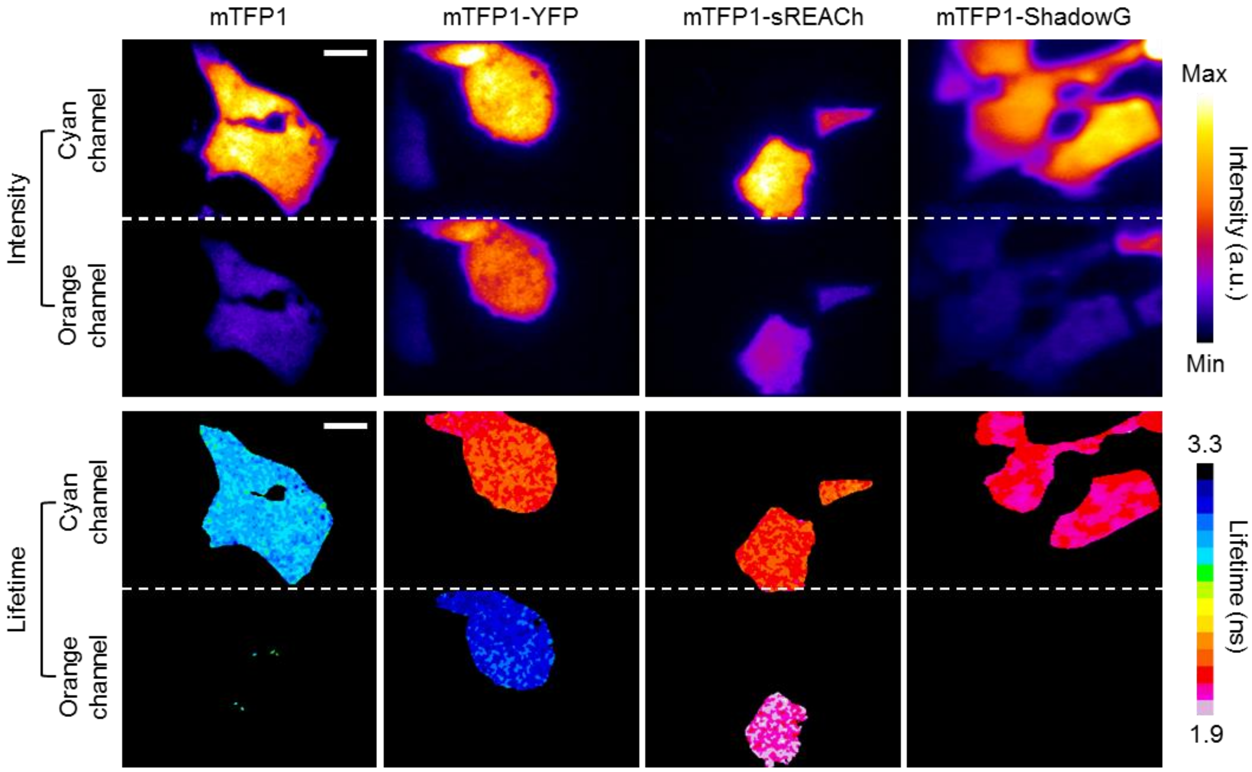

<u>Supplementary Figure 3: ShadowG is an effective FRET acceptor for mTFP1 minimizing its spectral bleed-through in the orange channel.</u>

Representative fluorescence intensity and lifetime images of U2OS cells expressing mTFP1, mTFP1-YFP, mTFP1-sREACh and mTFP1-ShadowG alone acquired on dual color FLIM system. Average fluorescence lifetimes from left to right; Cyan channel: τ_{mTFP1}=2.62±0.03ns (n=28), $\tau_{mTFP1\text{-}EYFP}$=2.02±0.04ns (n=15), $\tau_{mTFP1\text{-}sREACh}$=2.05±0.07ns (n=29), and $\tau_{mTFP1\text{-}ShadowG}$=2.14±0.05ns (n=21). Orange channel: $\tau_{mTFP1\text{-}EYFP}$=2.95±0.04ns (n=10), $\tau_{mTFP1\text{-}sREACh}$= 1.83±.10ns (n=3) corresponding to the EYFP and sREACh fluorescence lifetime contributions. Note that no fluorescence lifetime was recovered for mTFP1-ShadowG and mTFP1, as already seen in figure S1. Also, for similar mTFP1 fluorescence intensity levels in all conditions (top row), spectral bleed-through was higher in mTFP1-YFP, mTFP1-sREACh conditions while mTFP1-ShadowG was identical to mTFP1 alone. Fluorescence lifetimes were calculated as a mean±SD. (n) indicates number of cells from at least 3 independent experiments. Scale bar = 10 µm.

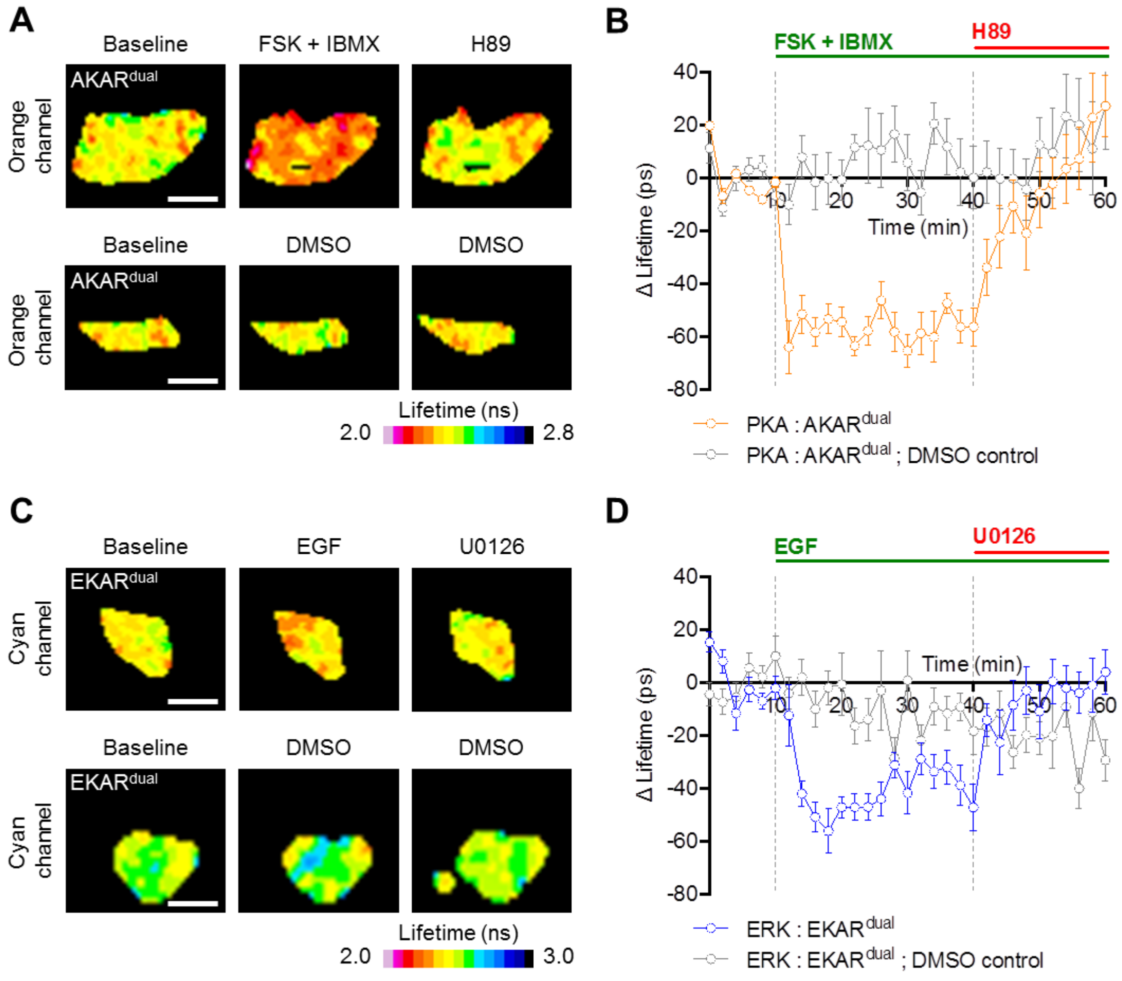

Supplementary Figure 4: AKAR^{dual} and EKAR^{dual} display expected reference experiment profiles with dual color FLIM system.

Kinase activity profiles from HeLa cells expressing either AKAR^{dual} (A) or EKAR^{dual} (C) and subjected to reference biosensing experiments were recorded single wavelength dual color FLIM system. (A) Representative fluorescence lifetime images AKAR^{dual} in the orange channel during: baseline (left top and bottom), upon Fsk/IBMX treatment (top middle), and H89 treatment (top right) or DMSO (bottom middle and right) as control. (B) The graph represents the mean AKAR^{dual} ΔLifetime along time (60min) during baseline (ΔLifetime of 0±2ps over 10min), upon activation by Fsk/IBMX (ΔLifetime of -46±3ps over 30min) and H89 inhibition (ΔLifetime of 1±5ps over 20min) phases (orange, n=7), and baseline followed by DMSO treatment (ΔLifetime of 10±2ps over 60min) as control (grey n=10). (C) Representative fluorescence lifetime images EKAR^{dual} in the cyan channel during: baseline (left top and bottom), upon EGF treatment (top middle), and U0126 treatment (top right) or DMSO (bottom middle and right) as control. (D) The graph represents the mean EKAR^{dual} ΔLifetime along time (60min) during the baseline (ΔLifetime of 0±2ps over 10min), upon activation by EGF (ΔLifetime of -40±2ps over 30min) and U0126 inhibition (ΔLifetime of -6 ±3ps over 20min) phases (blue, n=19), and baseline followed by DMSO treatment (ΔLifetime of -10±2ps over 60min) as control (grey n=12). [DMSO]=1/1000, [Fsk]=12.5µM, [IBMX]=75µM, [H89]= 20µM, [EGF]=100ng/ml, and [U0126]= 20 µM. Fluorescence lifetimes curves and means were calculated as a mean±SEMs. (n) indicates number of cells from at least 3 independent experiments. Scale bar =10 µm.

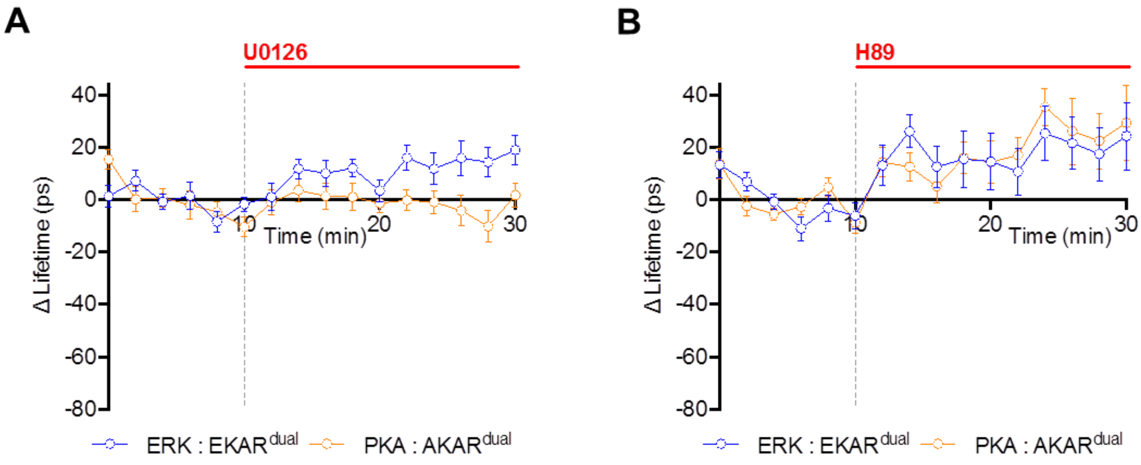

<u>Supplementary Figure 5: Basal activation level of PKA and ERK kinase.</u>
Graphs represent mean $EKAR^{dual}$ and $AKAR^{dual}$ ΔLifetime along time (40min) during baseline (mean ΔLifetime over 10min of 0±2ps for $EKAR^{dual}$ and $AKAR^{dual}$ in A and B) and U0126 or H89 inhibition (mean ΔLifetime over 20min of 12±2ps for $EKAR^{dual}$ and -1±2ps for $AKAR^{dual}$ in A and of 18 ±3ps for $EKAR^{dual}$ and 19±3ps for $AKAR^{dual}$ in B) phases. U0126 (n=19), H89 (n=11). [H89]= 20µM and [U0126]= 20 µM. Fluorescence lifetime curves were calculated as a mean±SEMs. (n) indicates the number of cells from at least 3 independent experiments.

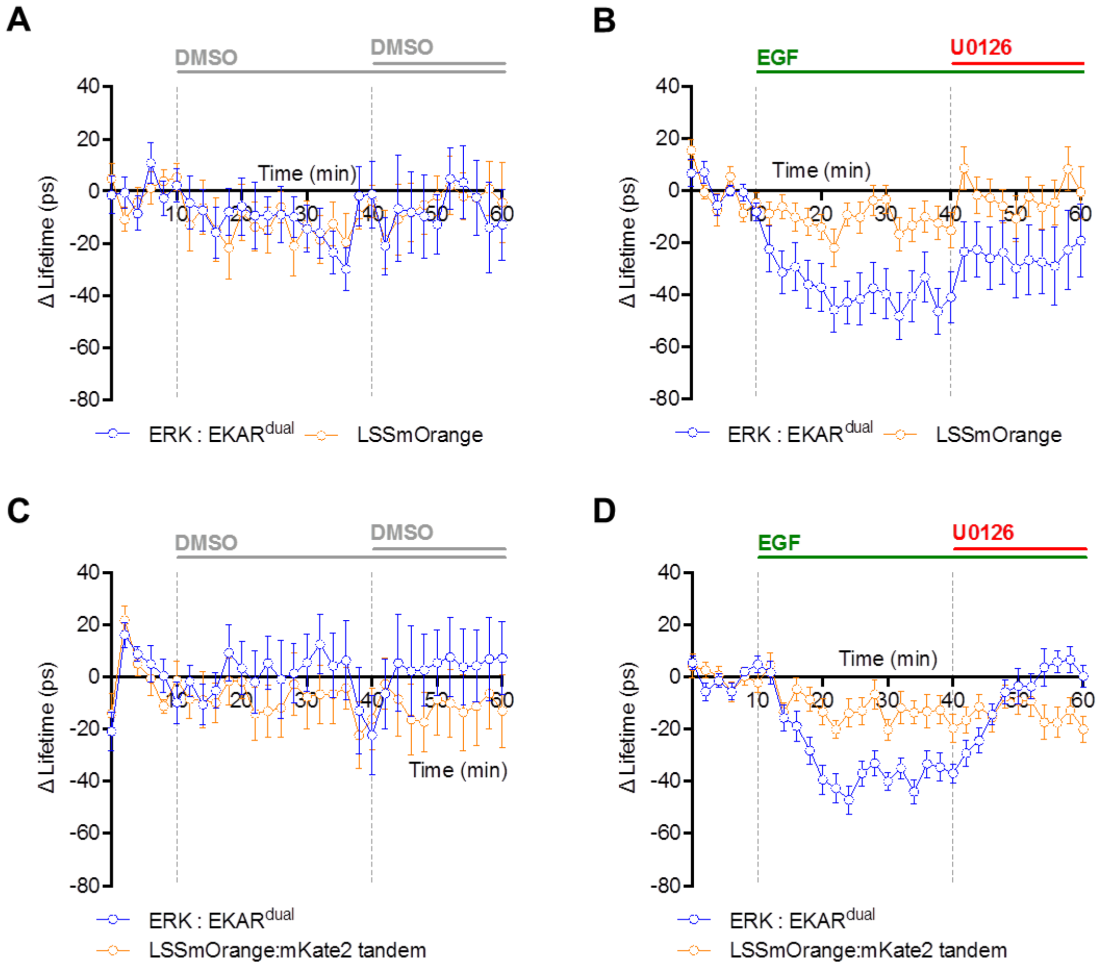

Supplementary Figure 6: No spectral bleed-through in the orange channel.

Graphs represent the mean LSSmOrange (orange curve) and EKAR^{dual} (blue curve) ΔLifetime along time (60min) during: (A) baseline followed by DMSO treatment (mean ΔLifetime of -8±2ps for LSSmOrange and -8±2ps for EKAR^{dual} over 60min) (n=10) and (B) baseline (mean ΔLifetime of -3±2ps for LSSmOrange and -2±2ps for EKAR^{dual} over 10min) upon activation by EGF (mean ΔLifetime of -10±1ps for LSSmOrange and -39±2ps for EKAR^{dual} over 30min) and U0126 treatment (mean ΔLifetime of -1±3ps for LSSmOrange and -25±4ps for EKAR^{dual} over 20min) phases (n=23).

Graphs represent the mean LSSmOrange-mKate2 (orange curve) and EKAR^{dual} (blue curve) ΔLifetime along time (60min) during: (C) baseline followed by DMSO treatment (mean ΔLifetime of -8±2ps for LSSmOrange and 1±2ps for EKAR^{dual} over 60min) (n=9) and (D) baseline (mean ΔLifetime of 0±2ps for LSSmOrange-mKate2 and EKAR^{dual} over 10min) upon activation by EGF (mean ΔLifetime of -12±1ps for LSSmOrange-mKate2 and -32±1ps for EKAR^{dual} over 30min) and U0126 treatment (mean ΔLifetime of -13±2ps for LSSmOrange-mKate2 and -6±2ps for EKAR^{dual} over 20min) phase (n=26).

[DMSO]=1/1000, [EGF]=100ng/ml and [U0126]=20µM. Fluorescence lifetime curves and means were calculated as mean±SEMs. (n) indicates number of cells from at least 3 independent experiments.

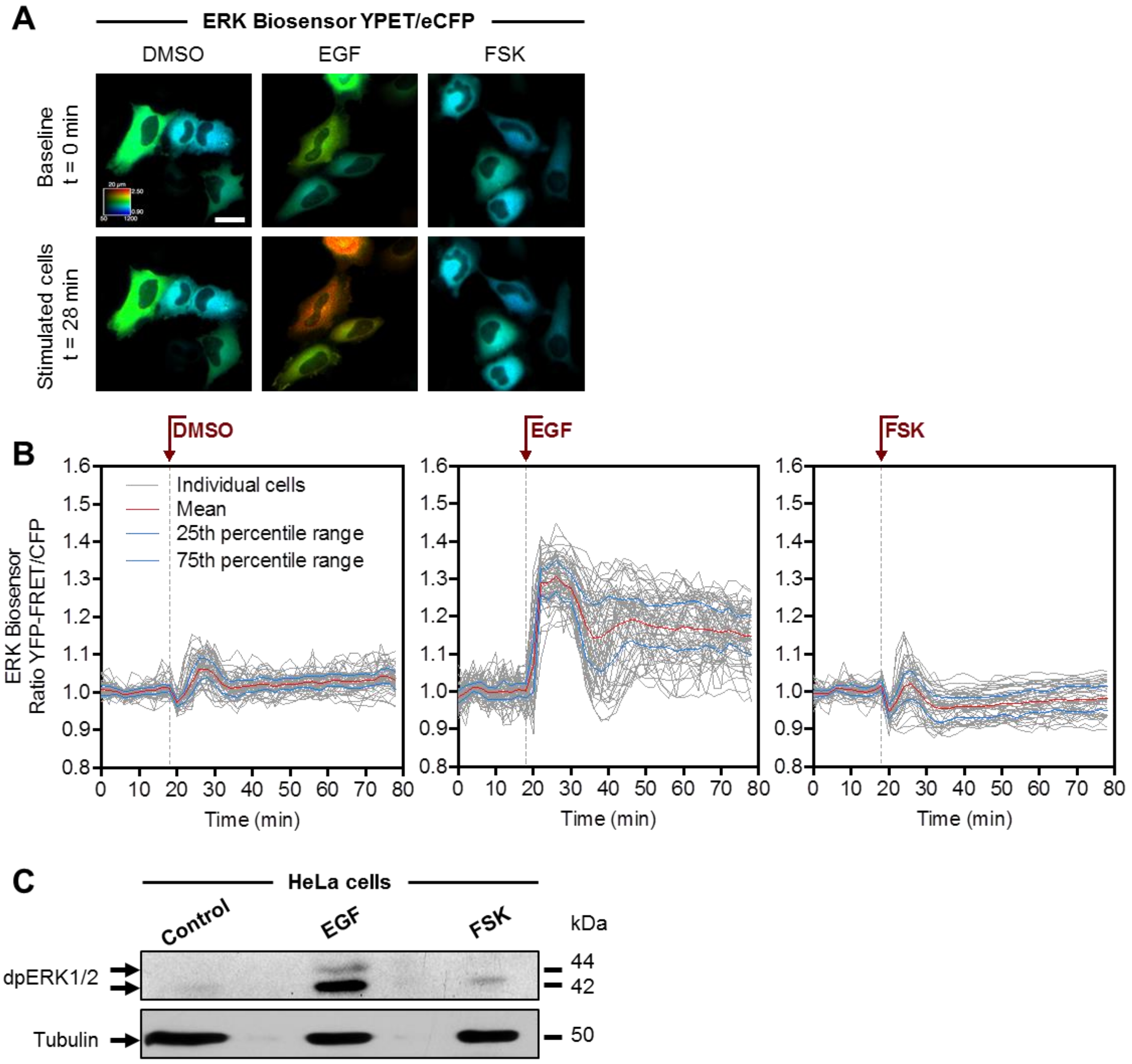

__Supplementary Figure 7: Fsk-mediated PKA activation in HeLa cells does not lead to MAPK/ERK1&2 activation.__

(A). Ratiometric CFP/YFP images of HeLa cells expressing a new optimized version of ERK biosensor (YPET/eCFP) (unpublished) before (t = 0min) and 10 min after treatments addition: DMSO (1/1000e, Left), EGF (100ng/ml, Middle) and Fsk (12.5µM, Right) (28 min after the onset of the experiment). Scale bars, 20µm. (B). ERK response to different treatment conditions, as measured by a new optimized version of ERK biosensor. The graphs plot FRET/CFP intensity ratio of EKAR over time for the different treatment conditions: Left, DMSO (n=49); Middle, EGF (n=60); Right, Fsk (n=44). Normalized FRET values are displayed in two ways: cell trace for each individual cell (grey) overlaid with the population mean (red) and the 25th and 75th percentile values (blue). Data are shown as mean ± SEM. (n) indicates number of cells from at least 3 independent experiments. (C) Cells were serum-starved for 12h with 0.1% serum containing medium and then stimulated with DMSO as a control (1/1000), Fsk (12,5 µM) and EGF (100ng/mL) for 10 min. Cellular lysates were analyzed by immunoblot with antibodies rat anti-TUBA1A clone YL1/2 (1/5000, EMD Millipore, MAB1864), mouse anti-MAP Kinase, activated (1/1000, Sigma Aldric,h M8159) and 1/5000 dilution of horseraldish peroxidase-conjugated anti-mouse or anti-rat antibodies for 1h (Jackson ImmunoResearch Laboratories, 315-035-045 and Bethyl Laboratories, A110-105P).

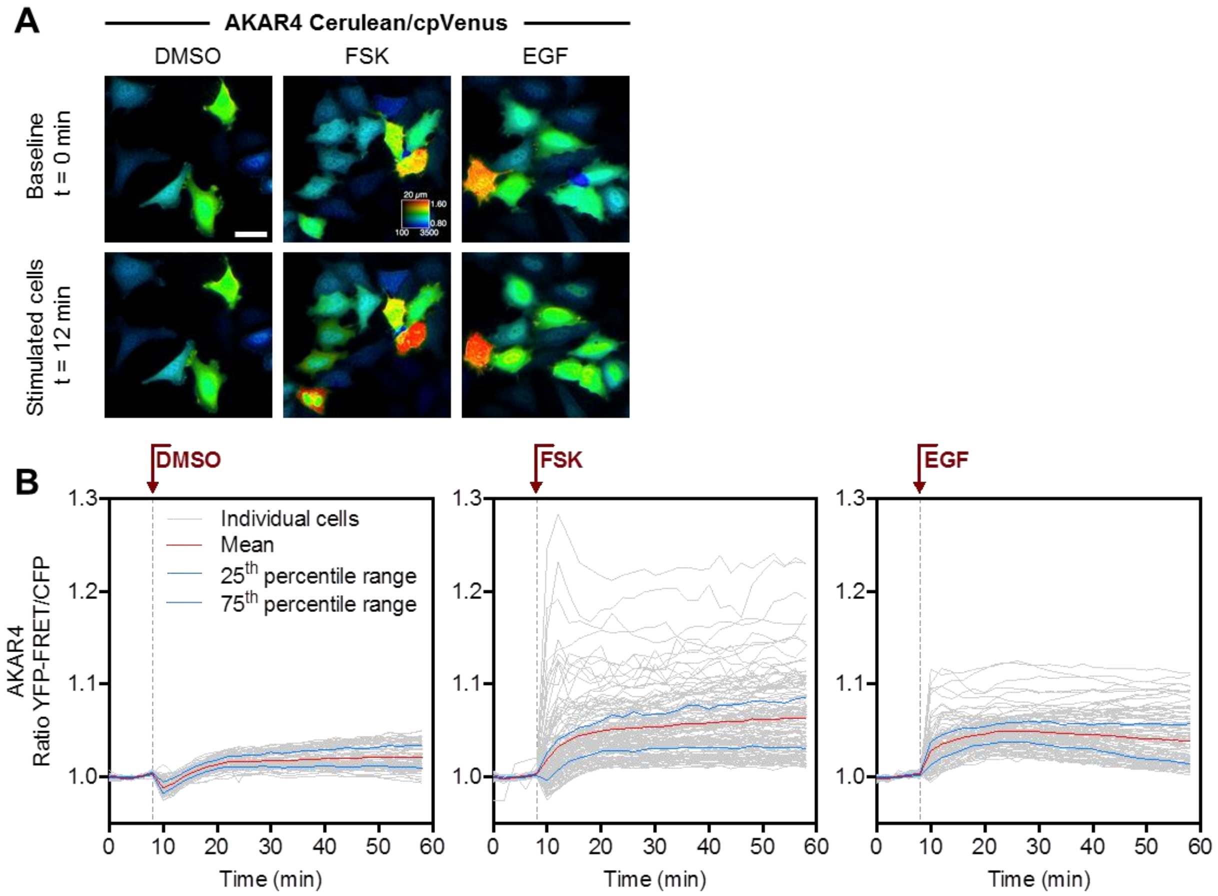

Supplementary Figure 8: EGF-mediated PKA activation in HeLa cells confirmed in single biosensor experiments using ratiometric FRET measurements.

(A). Ratiometric CFP/YFP images of HeLa cells expressing the original AKAR4 biosensor (Cerulean/cpVenus) before (t = 0min) and 4 min after treatments addition: DMSO (1/1000e, Left), Fsk (12.5µM, Middle) and EGF (100ng/ml, Right) (12 min after the onset of the experiment). Scale bars, 20µm. (B). PKA response to different treatment conditions, as measured by AKAR4. The graphs plot FRET/CFP intensity ratio of AKAR4 over time for the different treatment conditions: Left, DMSO (n=80); Middle, Fsk (n=134); Right, EGF (n=105). Normalized FRET values are displayed in two ways: cell trace for each individual cell (grey) overlaid with the population mean (red) and the 25th and 75th percentile values (blue). Data are shown as mean ± SEMs. At least 3 independent experiments were performed. (n) indicates number of cells from at least 3 independent experiments.
